# Influence of Antimicrobial Sealing Gels on Reverse Torque Values of Implant–Abutment Screws Following Thermomechanical Loading: A Comparative In Vitro Study

**DOI:** 10.3390/polym18151834

**Published:** 2026-07-27

**Authors:** Zeynep Irkeç, Ayben Şentürk, Kaan Orhan

**Affiliations:** 1 Department of Prosthetic Dentistry, Faculty of Dentistry, Lokman Hekim University, Ankara 06510, Turkey; 2 Department of Prosthetic Dentistry, Faculty of Dentistry, Ankara University, Ankara 06100, Turkey; aybensenturk@ankara.edu.tr; 3 Department of Dentomaxillofacial Radiology, Faculty of Dentistry, Ankara University, Ankara 06100, Turkey; knorhan@ankara.edu.tr; 4 Department of Oral Radiology, School and Hospital of Stomatology, Cheeloo College of Medicine, Shandong University, Jinan 250012, China

**Keywords:** dental implants, screw loosening, torque, chlorhexidine, silicones

## Abstract

Background: Abutment screw joint stability is essential for the long-term success of implant-supported restorations. However, the effects of antimicrobial agents on implant–abutment screw mechanics remain unclear. This in vitro study evaluated the effects of chlorhexidine and silicone-based sealing gels on reverse torque values (RTVs) following thermomechanical loading. Methods: Forty-five Straumann implant analog–abutment assemblies were allocated to three groups (*n* = 15 per group): untreated control, 2% chlorhexidine gel, and a silicone-based gel (Flow.sil) applied to the abutment screw threads prior to tightening. Cemented standardized monolithic zirconia crowns were subjected to thermomechanical aging (750,000 cycles, 100 N, 2 Hz, 5–55 °C). The mechanical performance of the screw joint was assessed using RTVs, whereas calculated torque loss percentages were reported as a complementary outcome to facilitate the clinical interpretation of the reverse torque findings. Data were analyzed using Shapiro–Wilk and Levene’s tests, one-way analysis of variance (ANOVA), and Tukey’s HSD test (α = 0.05). Results: Significant differences among groups were observed for post-aging RTVs (*p* < 0.001). The control group exhibited the highest RTVs (33.03 ± 6.57 Ncm), suggesting the most favorable post-aging reverse torque performance. RTVs were lower in the chlorhexidine (25.29 ± 4.78 Ncm) and Flow.sil (23.16 ± 7.39 Ncm) groups. Although the sealing agents did not differ significantly, chlorhexidine yielded numerically higher RTVs than Flow.sil. Conclusions: Under the present experimental conditions, direct application of antimicrobial and silicone-based sealing agents to the abutment screw threads was associated with lower RTVs than the untreated control condition. Accordingly, the potential mechanical effects of these materials should be considered when selecting screw-sealing protocols.

## 1. Introduction

In implant-supported prostheses, the abutment screw represents the most critical component of the system [1]. Screw loosening and abutment or implant fracture remain among the most frequently reported mechanical failures [2,3]. Screw loosening is particularly common in single-implant restorations, with reported incidences ranging from 12.7% to 43% [4,5], whereas screw fractures occur less frequently, affecting approximately 3.5% of cases during long-term follow-up [6]. Alongside these complications, peri-implantitis associated with progressive crestal bone loss remains a major biological challenge affecting the long-term success of implant therapy [1,7]. The implant–abutment interface (IAI), where the implant and abutment are connected by the abutment screw, has been identified as a potential pathway for oral fluid and microbial penetration, thereby contributing to the development of peri-implant disease [7].

The IAI may be exposed to biological fluids, such as saliva and blood, as well as clinically applied agents, including fluoridated artificial saliva and chlorhexidine [1]. A previous study has shown that saliva contamination may increase preload levels [8], while fluoridated artificial saliva has been associated with higher reverse torque values (RTVs) [9]. Various sealing materials, including silicone-based sealants, have been proposed to reduce microleakage at the IAI and potentially limit biological complications [3,7]. Although numerous studies have investigated the effects of antimicrobial sealing agents on microleakage [10,11,12], evidence regarding their influence on the screw joint stability (SJS) remains unclear. Micarelli et al. (2013) found no significant effect of chlorhexidine gel application within the screw channel on abutment screw RTVs [13], whereas Gumus et al. (2014) reported lower RTVs following this procedure [14]. The highest RTVs were recorded in the chlorhexidine group, whereas no significant differences were detected between blood and fluoridated artificial saliva contamination in a study by Koosha et al. [1]. Collectively, these findings remain inconclusive due to the limited and conflicting evidence, highlighting the need for further investigation into the effects of antimicrobial sealing agents on SJS.

During tightening, elastic deformation of the screw followed by elastic recovery generates a clamping force that draws the implant and abutment components together. The preload generated through this mechanism is essential for maintaining SJS and minimizing the risk of screw loosening [2]. For optimal SJS under dynamic loading, preload is generally recommended to range between 60% and 75% of the screw’s yield strength [15]. Beyond tightening torque, preload is influenced by several variables, including the elastic modulus of the screw, the coefficient of friction, and lubrication conditions that may alter their interaction [2,16]. Chlorhexidine-containing sealing gels have been proposed to provide a disinfectant barrier, whereas silicone-based gels have been advocated to fill the implant–abutment gap and reduce bacterial leakage [3]. However, these agents may create a distinct tribological environment at the IAI, potentially modifying frictional behavior and surface interactions, which may affect long-term SJS [3,16].

Friction is known to increase with surface roughness and hardness while decreasing with tightening speed and lubrication [15]. Reduced friction may facilitate a more efficient conversion of the applied torque into preload, thereby promoting higher initial preload values [16]. Nevertheless, the long-term success of the implant–abutment connection depends not only on the generation of adequate preload but also on its maintenance within the elastic limits of the screw [3]. Accordingly, the recommended tightening torque is generally intended to generate preload levels that do not exceed approximately 80% of the screw’s elastic limit. Exceeding this threshold may result in plastic deformation, leading to preload loss, screw loosening, and ultimately screw fracture [17]. However, the influence of sealing agents on preload generation and maintenance remains unclear. Rathe et al. (2021) found that neither silicone-based nor chlorhexidine-containing gels had a significant influence on preload values [3]. Taken together, the tribological mechanisms operating at the IAI are complex and not yet fully understood [15]. Consequently, further investigation is warranted to clarify the effects of sealing agents and lubrication conditions on screw joint mechanics under dynamic loading.

In a previous study, wrapping the screw with polytetrafluoroethylene (PTFE) was associated with reduced preload loss, which was attributed to its potential stress-absorbing effect [18]. More recently, an investigation evaluating the influence of screw access channel filling materials on post-aging RTVs reported the highest RTVs in abutments sealed with polyvinyl siloxane putty, particularly in rigid monolithic zirconia restorations [19]. These findings suggest that silicone-based materials may alter mechanical effects beyond their sealing function. Therefore, evaluating the direct application of silicone-containing agents at the IAI may provide further insight into their potential influence on SJS.

RTV is widely accepted as an indirect indicator of residual preload and is commonly used to evaluate SJS following mechanical fatigue [20]. The present in vitro study evaluated the influence of antimicrobial sealing gels applied to implant–abutment screws on RTVs following thermomechanical loading. Post-aging RTVs served as the primary outcome measure, whereas calculated torque loss percentages were included to provide complementary clinical interpretation. It was hypothesized that the silicone-based sealing material would result in higher RTVs following thermomechanical loading owing to its potential stress-absorbing and load-distributing properties. In contrast, the chlorhexidine gel and untreated control groups were expected to exhibit lower RTVs, with no significant difference anticipated between these two conditions.

## 2. Materials and Methods

This in vitro investigation was carried out at the Faculty of Dentistry, Ankara University, Ankara, Turkey. Since the study did not involve human participants, animal subjects, or biological tissues, ethical approval was not required.

### 2.1. Study Design and Sensitivity Analysis

A total of 45 implant–abutment screw assemblies were included in the study, with 15 specimens allocated to each of the three independent groups. A sensitivity power analysis was conducted using G*Power software (version 3.1.9.7; Heinrich Heine University, Düsseldorf, Germany) for a fixed-effects omnibus one-way ANOVA. With a total sample size of 45 specimens, three independent groups, an alpha level of 0.05, and 80% power, the minimum detectable effect size was Cohen’s *f* = 0.48, corresponding to η^2^ = 0.187. Therefore, the study was adequately sensitive to detect group effects of approximately this magnitude or greater.

### 2.2. Preparation of Implant Analog–Abutment Specimens

A total of 45 implant analog–abutment specimens were prepared and assigned to three experimental groups (*n* = 15) according to the sealing protocol evaluated in the study. Randomized allocation was performed to ensure unbiased group assignment, as all specimens consisted of identical implant analogs, abutments, and manufacturer-provided screws, thereby providing a standardized geometric and mechanical configuration across the experimental groups. Straumann NC Bone Level implant analogs (11 mm length; Ref. 025.2101; titanium–aluminum–niobium alloy, Ti-6Al-7Nb; Institut Straumann AG, Basel, Switzerland) and matching titanium-alloy NC cementable abutments (3.5 mm diameter, 5.5 mm abutment height, and 2 mm gingival height; Institut Straumann AG, Basel, Switzerland) were used throughout the study. The implant analog–abutment complexes featured an internal connection geometry designed to provide standardized mechanical engagement during testing. Original manufacturer-provided abutment screws were used in all specimens. Prior to specimen preparation, the abutments were connected to the corresponding implant analogs and lightly hand-tightened to ensure complete seating and facilitate handling during the embedding procedure.

**Figure 1:**
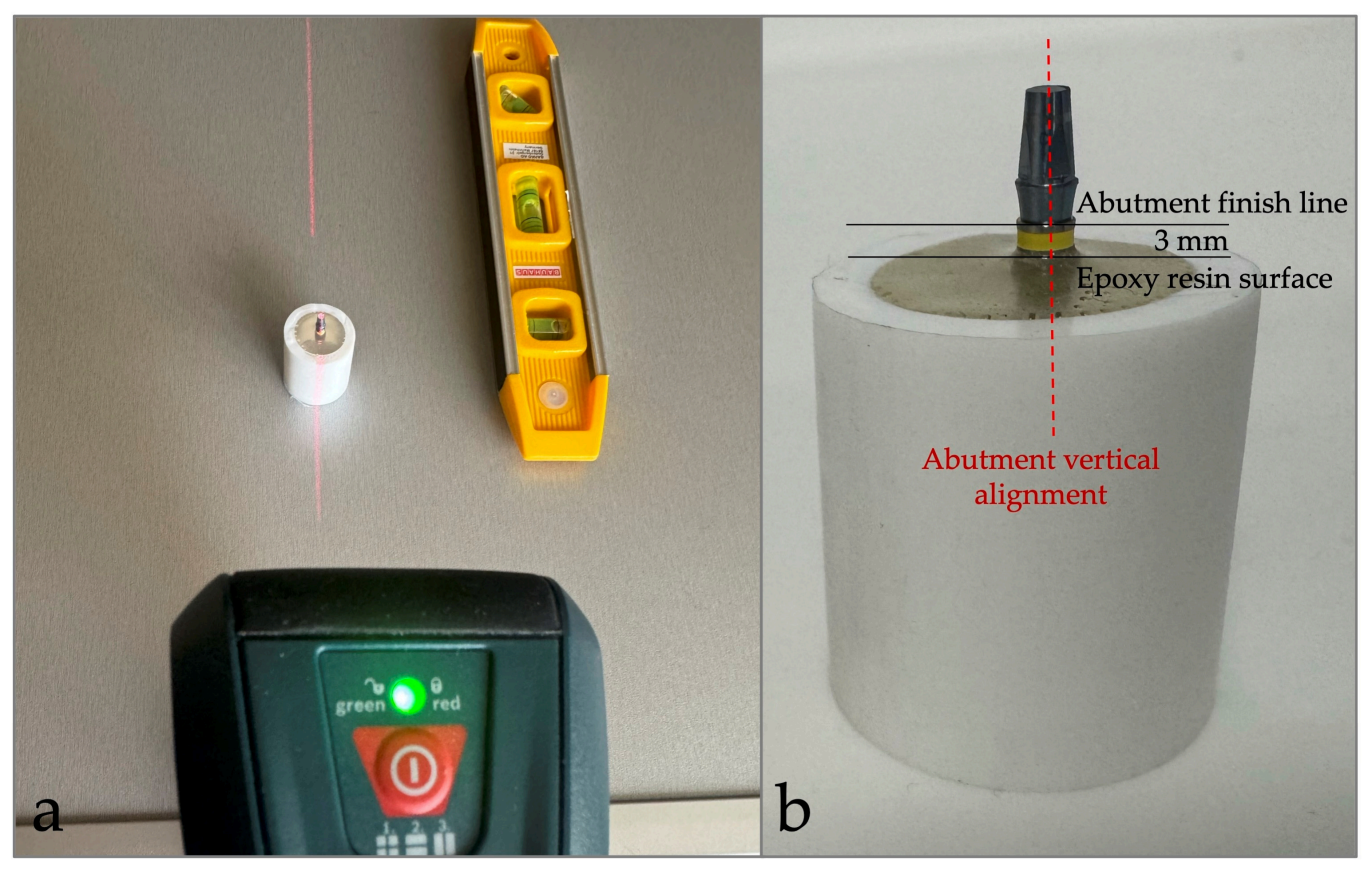
Implant analog–abutment specimen preparation: (**a**) verification of perpendicular alignment during embedding using a line laser level and a spirit level; (**b**) standardized specimen configuration showing the 3-mm distance between the abutment finish line and the epoxy resin surface and the vertical alignment of the abutment.

Standardized cylindrical molds fabricated from polypropylene random copolymer were used for specimen preparation. Each mold measured 30 mm in height, with an outer diameter of 25 mm and an inner diameter of 18 mm, corresponding to the dimensions required for the chewing simulator. The implant analog–abutment specimens were embedded in an epoxy resin model material (PL-2 and PLH-2; Vishay Precision Group Inc., Raleigh, NC, USA). During specimen fabrication, a line laser level (Professional GLL 2; Robert Bosch Power Tools GmbH, Stuttgart, Germany) was used to align the long axis of each abutment perpendicular to the horizontal plane. The accuracy of the horizontal reference plane was verified using a spirit level prior to resin polymerization to ensure consistent specimen positioning (Figure 1a).

During embedding, the implant analogs were positioned so that a 3-mm vertical distance was maintained between the abutment finish line and the resin surface (Figure 1b). This distance was verified using a digital caliper to ensure consistent specimen positioning and to simulate the 3-mm bone loss (worst-case bone support) condition described in ISO 14801:2016 [21]. The resin was then allowed to polymerize at room temperature for 24 h before further procedures.

### 2.3. Sealing Gel Application and Screw Tightening

**Figure 2:**
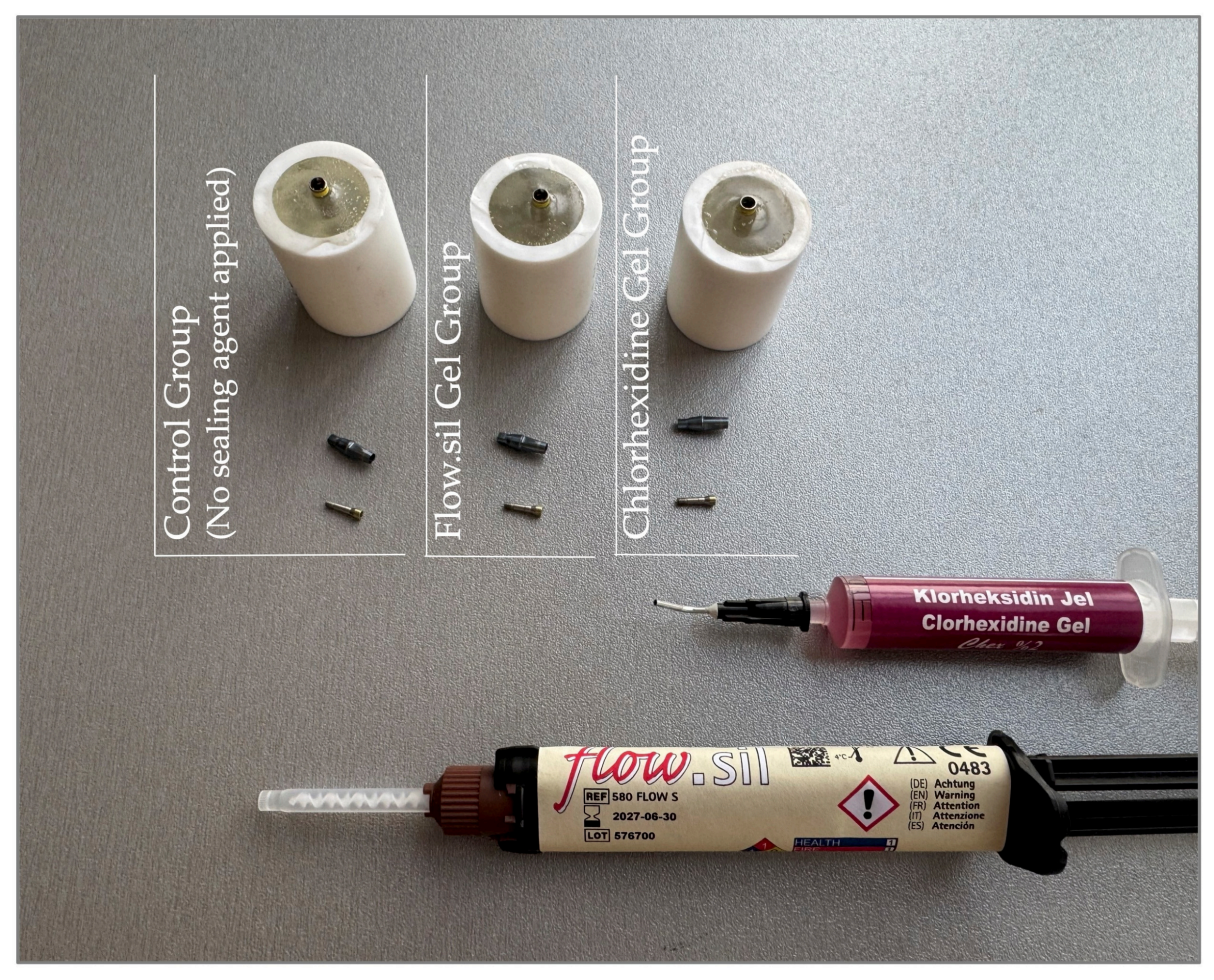
Experimental groups and sealing agents used in the study.

Following specimen preparation, the abutment screws, which had been previously hand-tightened only to facilitate specimen handling during embedding, were removed from the implant analog–abutment assemblies. Prior to final torque application, no material was applied to the screws in the control group. In the chlorhexidine group, a 2% chlorhexidine-based gel (Best Chex; Spident Co., Ltd., Incheon, South Korea) was applied to the abutment screw threads, whereas a silicone-based sealing gel (Flow.sil, bredent medical GmbH & Co. KG, Senden, Germany) was applied to the screw threads in the third group according to the manufacturer’s recommendations (Figure 2).

**Figure 3:**
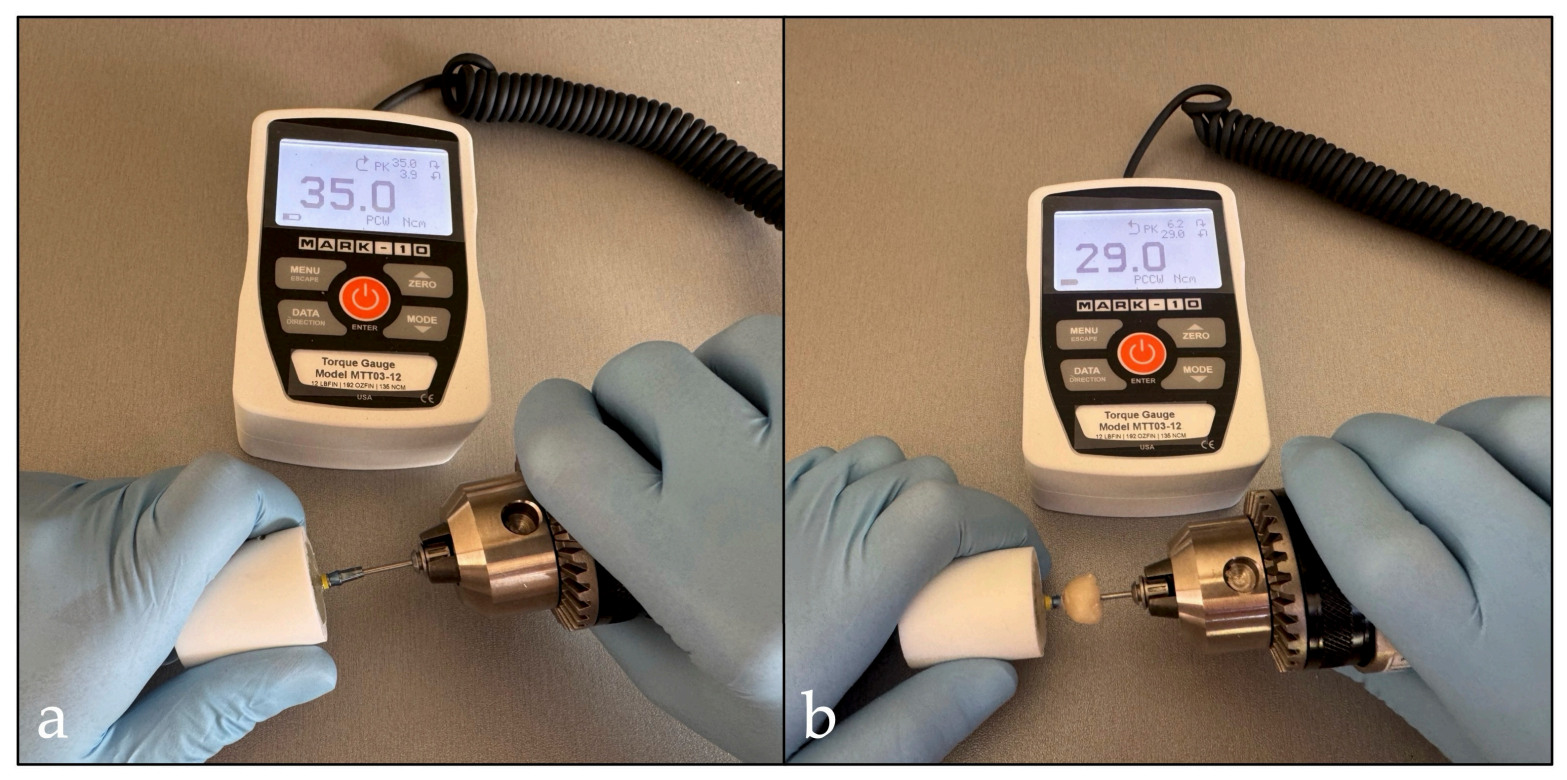
Torque measurement procedures: (**a**) tightening of the abutment screw to 35 Ncm using a calibrated digital torque gauge operating in peak clockwise mode; (**b**) recording of the reverse torque value (RTV) after thermomechanical aging using the same device in peak counterclockwise mode.

In both experimental groups, the gels were applied directly from the manufacturer-provided dispensing tip as a carefully applied thin layer over the entire threaded portion of each abutment screw to provide consistent coverage of the screw threads and thread grooves. All gel applications were performed by the same experienced operator. Given that the primary outcome of the study was reverse torque performance, the tested materials were intentionally applied to the abutment screw threads before final tightening to allow direct interaction with the screw–implant interface. Immediately following gel application, the abutment screws were initially hand-tightened to facilitate gradual seating. This procedure allowed proper distribution of the chlorhexidine gel and minimized excessive resistance that could arise from the time-dependent hardening behavior of the silicone-based sealing gel during subsequent torque application. Excess gel that overflowed during this procedure was carefully removed using a microbrush before final torque application. The abutment screws were then tightened to 35 Ncm using a calibrated digital torque gauge (MTT03-12, TT03 Series; Mark-10 Corporation, Copiague, NY, USA; measuring range: 0–135.6 Ncm [12 lbf·in], accuracy: ±0.5% of full scale, resolution: 0.01 Ncm) (Figure 3a).

To minimize preload loss associated with embedment relaxation, a second torque application at the same value was performed after a 10-min interval [22]. All tightening procedures were carried out by a single experienced operator using the same calibrated device to ensure procedural standardization and reduce operator-related variability among specimens. Immediately after the second torque application, the specimens proceeded directly to the crown cementation procedures without any additional waiting period to minimize any potential influence of elapsed time on the RTV measurements.

### 2.4. Monolithic Zirconia Crown Fabrication

A mandibular first molar geometry was deliberately selected to reproduce the direction and distribution of occlusal forces encountered under clinical conditions and to allow vertical load application in the chewing simulator in a manner consistent with physiological loading conditions. Monolithic zirconia crowns (Sigma Monolithic Zirconia; Sigmadent, Istanbul, Türkiye) were designed for all specimens using dental CAD software (Exocad GmbH, Darmstadt, Germany; https://exocad.com; accessed on 18 April 2025).

**Figure 4:**
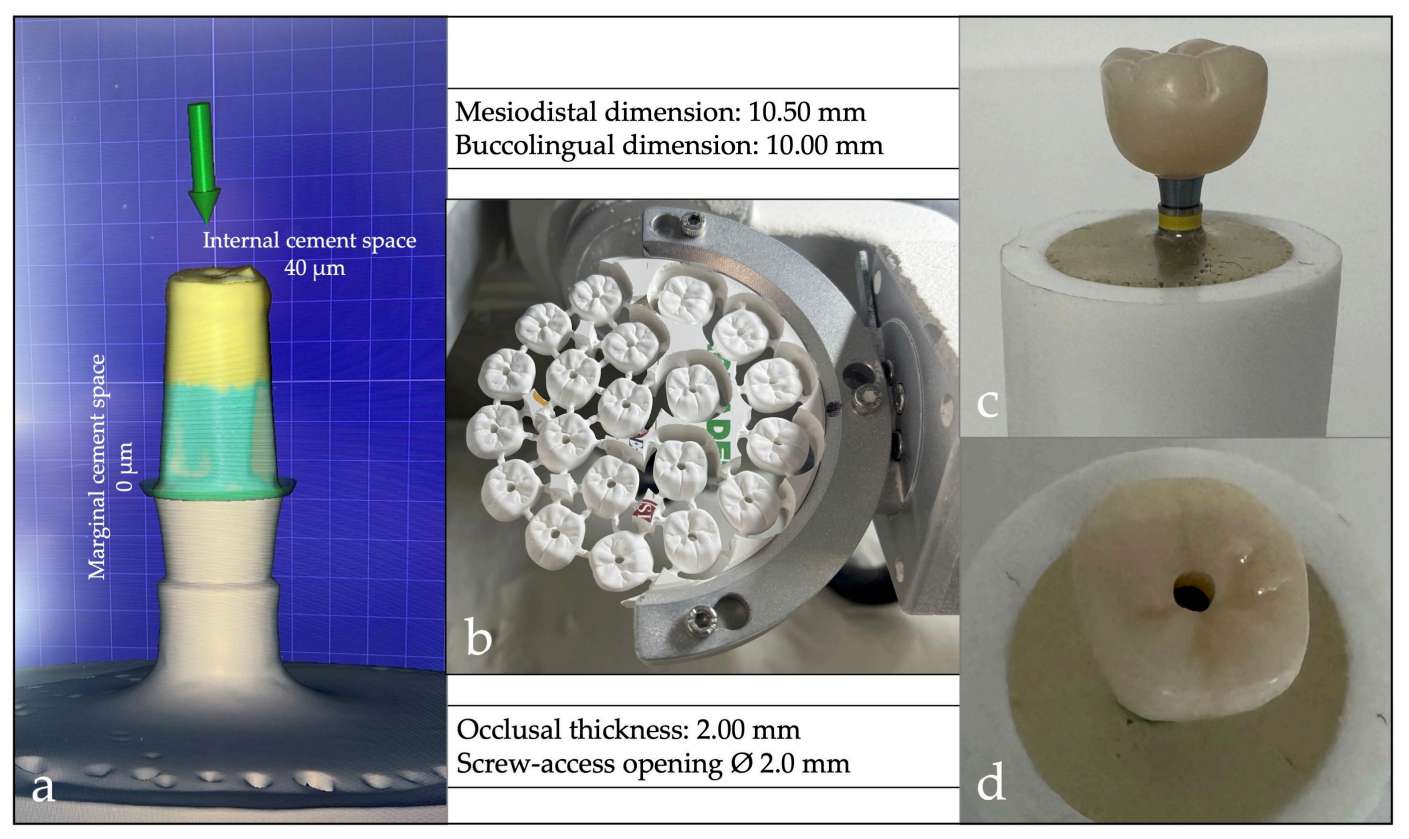
Design and fabrication workflow of the monolithic zirconia crowns: (**a**) CAD design illustrating the standardized internal and marginal cement spaces; the arrow indicates the insertion path of the crown, and the colors represent the default CAD software visualization; (**b**) zirconia crowns after milling; (**c**) representative crown–abutment assembly; and (**d**) occlusal view demonstrating the screw-access opening.

The internal cement space was set at 40 μm, while the marginal cement space was maintained at 0 μm to enhance crown–abutment stability and reduce the potential for rotational movement during thermomechanical loading (Figure 4a).

To promote consistent load transfer during thermomechanical aging, the occlusal anatomy was designed with shallow fossae and reduced cusp inclinations. The crown designs were transferred to CAM software (WorkNC; Hexagon Manufacturing Intelligence, Saint-Aubin, France; https://hexagon.com; accessed on 18 April 2025) for milling using a 5-axis milling unit (Redon Hibrit; Redon Technology, Istanbul, Türkiye) operating under dry conditions (Figure 4b). A standardized 2 mm manufacturer-provided milling bur was used for fabrication of all crowns. Following milling, the zirconia crowns were sintered according to the manufacturer’s recommended firing protocol to achieve their final microstructural and mechanical properties before cementation procedures. The final crown design was standardized to provide mesiodistal and buccolingual dimensions of 10.5 mm and 10.0 mm, respectively, an occlusal thickness of 2.0 mm, and a centrally positioned 2.0-mm screw-access opening to facilitate subsequent reverse torque measurement (Figure 4c,d).

### 2.5. Screw Access Sealing and Crown Cementation

Prior to crown cementation, the screw-access channels were sealed using a two-layer PTFE tape protocol [19]. Initially, a thin PTFE layer was carefully packed over the screw head to provide precise isolation. Subsequently, an additional PTFE layer was condensed to act as a cushioning layer and facilitate access to the screw head during subsequent retrieval procedures. Its thickness was adjusted using a periodontal probe to maintain 2 mm of coronal space for composite resin placement. The remaining portion of the access cavity was restored with a light-cured nano-hybrid composite resin (Polofil NHT A2; VOCO GmbH, Cuxhaven, Germany) and polymerized for 20 s using an LED curing unit (Curing Pen-E, model C-004-1; Changzhou Sifary Medical Technology Co., Ltd., Changzhou, China) according to the manufacturer’s instructions.

The abutment surfaces were treated by airborne-particle abrasion using 50-μm aluminum oxide particles at 2 bar pressure for 10 s, applied perpendicularly from a distance of 10 mm with a laboratory sandblasting unit (Twin-Pen Sandblaster VI, model JG-218; Wuhan Jinguang Medical Technology Co., Ltd., Wuhan, China). Following surface treatment, the abutments were cleaned with alcohol and thoroughly air-dried. The intaglio surfaces of the monolithic zirconia crowns were subjected to the same airborne-particle abrasion protocol and subsequently conditioned with an MDP-containing primer (Z-Prime Plus; Bisco, Schaumburg, IL, USA). The primer was allowed to react for 60 s before excess solvent was evaporated with gentle air application for 10 s, in accordance with the manufacturer’s instructions.

Following surface conditioning, a dual-cure resin cement (SET PP; SDI Limited, Bayswater, Victoria, Australia) was applied to the internal surfaces of the zirconia crowns. Each crown was then seated onto its corresponding abutment using finger pressure by a single experienced operator until complete adaptation at the finish line was visually confirmed. Following crown seating, the cement was tack-cured for 2 s from both the cervical margins and the screw-access opening to facilitate removal of excess material. Excess cement was then carefully removed from these regions. Final light polymerization was subsequently performed for 20 s from each aspect of the restoration, including the screw-access opening, according to the manufacturer’s instructions. Subsequently, the screw-access channels were restored with the same light-cured nano-hybrid composite resin used for access channel sealing (Polofil NHT A2; VOCO GmbH, Cuxhaven, Germany) and polymerized using the previously described LED curing unit according to the manufacturer’s instructions. All specimens were subsequently stored at room temperature for 24 h to allow complete cement polymerization before thermomechanical aging procedures.

### 2.6. Thermomechanical Aging

**Figure 5:**
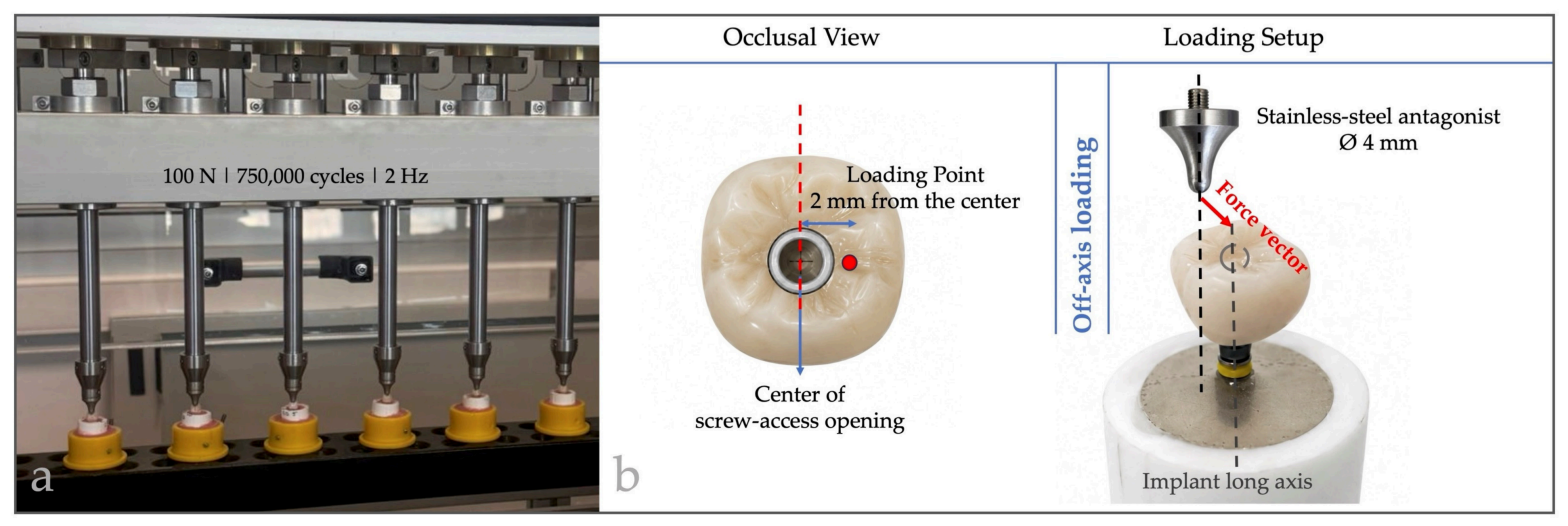
Thermomechanical aging setup and loading configuration: (**a**) computer-controlled chewing simulator used for thermomechanical aging (100 N, 750,000 cycles, 2 Hz); (**b**) schematic illustration of the experimental loading setup. The red dot indicates the loading point. In the rightmost image of panel (**b**), the circle represents the screw-access opening, the right dashed line represents the implant long axis, and the left dashed line indicates the loading direction of the indenter tip.

Following cementation, all specimens were subjected to thermomechanical aging using a computer-controlled chewing simulator (DentArge ACS 8; Analitik Medikal, Ankara, Turkey) (Figure 5a).

Cyclic loading was applied at 100 N for 750,000 cycles at a frequency of 2 Hz to simulate prolonged posterior occlusal function. Mechanical loading was delivered through a 4-mm-diameter stainless-steel antagonist under standardized conditions. The use of cemented monolithic zirconia crowns and the selection of a loading point positioned 2 mm away from the center of the screw-access opening to avoid direct contact with the composite-restored area were study-specific adaptations to the experimental setup. The resulting off-axis loading condition was based on the principles of ISO 14801:2016 [21] (Figure 5b). Simultaneously, thermal cycling was performed between 5 °C and 55 °C using the integrated thermocycling module of the chewing simulator, with a dwell time of 60 s at each temperature. Thermal and mechanical aging were applied concurrently throughout the entire aging procedure according to the chewing simulator’s integrated thermocycling protocol. The chewing simulator operates as a single, fully integrated thermomechanical unit that automatically synchronizes thermal cycling with mechanical loading, ensuring standardized and reproducible aging conditions for all specimens.

### 2.7. Recording of Reverse Torque Values

After thermomechanical aging, the composite restorations sealing the screw-access openings were carefully removed using a high-speed handpiece under water cooling and controlled pressure. Both PTFE layers were subsequently retrieved, exposing the screw head for reverse torque testing. RTVs were then recorded using the same calibrated digital torque gauge employed during the initial tightening procedures. Counterclockwise rotational force was applied until the onset of screw loosening, and the peak counterclockwise reverse torque value required to initiate screw movement was recorded in Ncm for each specimen (Figure 3b). All measurements were performed by the same operator under standardized testing conditions to ensure procedural consistency. Torque loss percentage was subsequently calculated for each specimen using the following formula:

$$Torque\;Loss\left(\%\right)=\frac{35-RTV}{35}\times 100$$

where 35 Ncm represented the initial tightening torque and RTV represented the reverse torque value measured after thermomechanical aging [7].

### 2.8. Statistical Analysis

All statistical analyses were performed using Python (version 3.11; Python Software Foundation, Wilmington, DE, USA) with the SciPy (version 1.11), NumPy (version 1.24), Pandas (version 2.0), and Matplotlib (version 3.7) libraries. Descriptive statistics were calculated for all outcome variables and reported as mean, standard deviation (SD), median, minimum–maximum values, and 95% confidence intervals (95% CIs). The normality of data distribution was assessed using the Shapiro–Wilk test, while homogeneity of variances was evaluated using Levene’s test. Since the assumptions of normality and homogeneity were satisfied, intergroup comparisons were performed using one-way analysis of variance (ANOVA). ANOVA results were reported as F-statistics, degrees of freedom (df), *p*-values, and effect sizes (η^2^). Effect sizes were interpreted according to Cohen’s criteria, where η^2^ values of 0.01, 0.06, and 0.14 corresponded to small, medium, and large effects, respectively. When statistically significant differences were identified, pairwise comparisons were conducted using Tukey’s honestly significant difference (HSD) post hoc test with adjustment for multiple comparisons. Mean differences, adjusted *p*-values, and 95% confidence intervals were reported. In addition, pairwise effect sizes were calculated using Cohen’s d and interpreted as negligible (<0.20), small (0.20–0.49), medium (0.50–0.79), large (0.80–1.19), or very large (≥1.20). Potential outliers were identified using the 1.5× interquartile range (IQR) criterion. In the absence of evidence of measurement error, all observations were retained for the primary analyses. To evaluate the robustness of the findings, sensitivity analyses were performed by repeating the ANOVA after exclusion of identified outliers. For all analyses, statistical significance was set at α = 0.05.

## 3. Results

No specimen failures, screw fractures, or component deformations were observed during thermomechanical aging. Descriptive statistics for RTVs and torque loss percentages are presented in Table 1.

**Figure 6:**
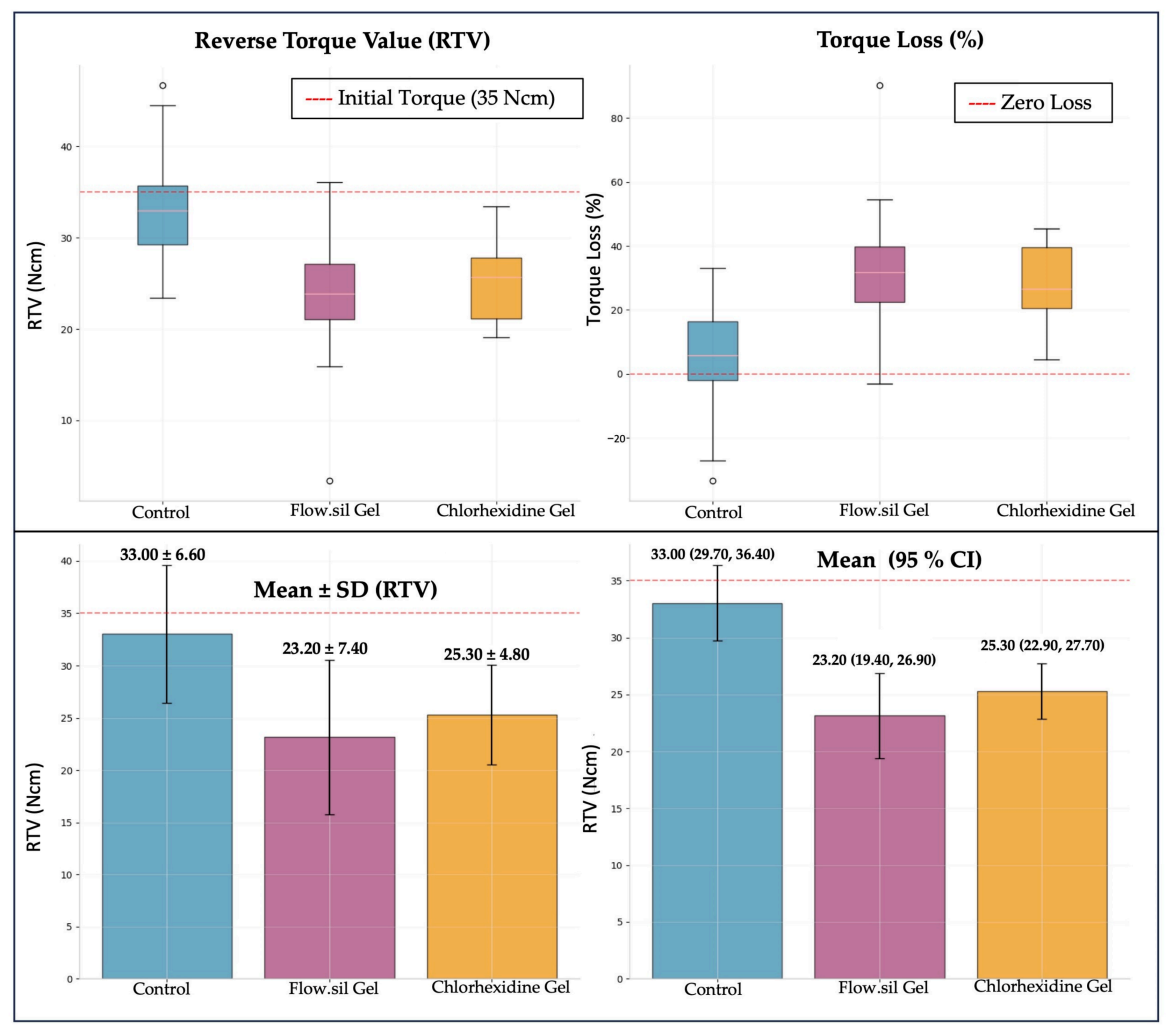
Reverse torque value (RTV) and torque loss (%) measurements following thermomechanical aging. The upper panels show boxplots of RTV and torque loss distributions for the Control, Flow.sil Gel, and Chlorhexidine Gel groups. Dashed red lines represent the initial tightening torque (35 Ncm) and zero torque loss, respectively. The lower panels present RTV data as mean ± standard deviation (SD) and mean with 95% confidence intervals (CI). Open circles indicate outlier values.

**Figure 7:**
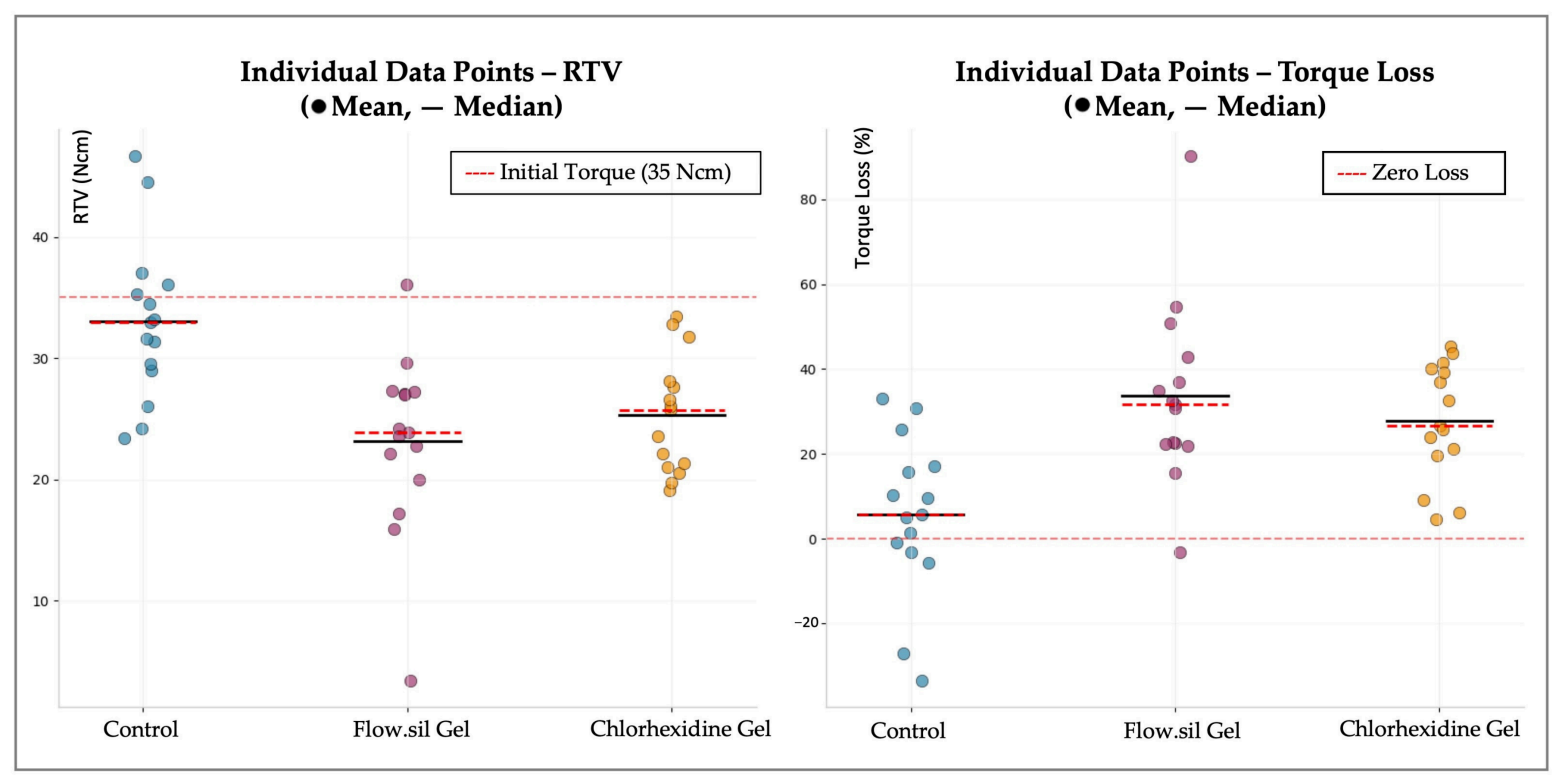
Individual reverse torque value (RTV) and torque loss (%) measurements following thermomechanical aging. Each point represents an individual specimen from the Control, Flow.sil Gel, and Chlorhexidine Gel groups (*n* = 15). Red dashed lines denote group means, and black solid lines indicate group medians. Reference dashed lines correspond to the initial tightening torque (35 Ncm) and zero torque loss (%), respectively.

The control group demonstrated the highest RTVs, with a mean value of 33.03 ± 6.57 Ncm (median: 33.00 Ncm; range: 23.40–46.70 Ncm; 95% CI: 29.70–36.35 Ncm). Lower RTVs were observed in the chlorhexidine gel group (25.29 ± 4.78 Ncm; median: 25.70 Ncm; range: 19.10–33.40 Ncm; 95% CI: 22.87–27.71 Ncm) and the Flow.sil gel group (23.16 ± 7.39 Ncm; median: 23.90 Ncm; range: 3.40–36.10 Ncm; 95% CI: 19.42–26.90 Ncm). For descriptive purposes, the corresponding torque loss percentages were 5.64 ± 18.77% in the control group, 27.75 ± 13.66% in the chlorhexidine gel group, and 33.83 ± 21.11% in the Flow.sil gel group (Figure 6 and Figure 7).

**Figure 8:**
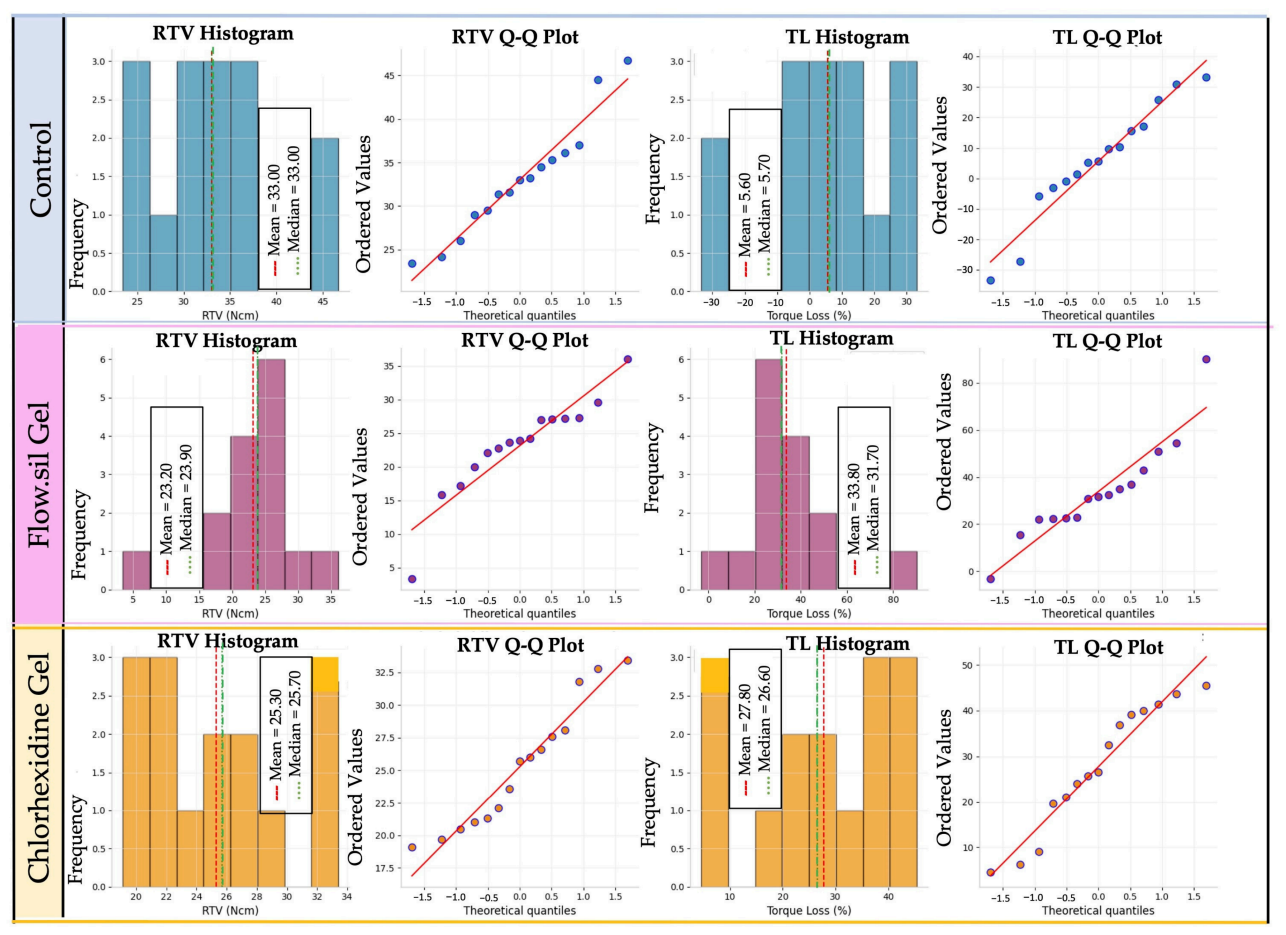
Histograms and Q–Q plots illustrating the distribution of reverse torque values and torque loss (%) data in the Control, Flow.sil Gel, and Chlorhexidine Gel groups. The red dashed and green dotted lines indicate the mean and median, respectively; dots in the Q–Q plots represent the ordered sample values plotted against theoretical quantiles, and the solid line denotes the reference line for a normal distribution. In the Control group, the mean and median lines overlap due to very close or identical values.

Normality assessment using the Shapiro–Wilk test confirmed that RTVs were normally distributed in all groups (Control: W = 0.946, *p* = 0.469; Flow.sil: W = 0.906, *p* = 0.117; Chlorhexidine: W = 0.925, *p* = 0.226). In addition, Levene’s test demonstrated homogeneity of variances among groups (F = 0.237, *p* = 0.790), supporting the use of parametric statistical analyses (Table 2a,b and Figure 8).

One-way ANOVA revealed a statistically significant effect of the experimental protocol on RTV following thermomechanical aging (F(2,42) = 10.06, *p* < 0.001, η^2^ = 0.324). The observed effect size indicated that 32.4% of the total variation in RTV was attributable to the experimental intervention, representing a large effect according to Cohen’s criteria (Table 3).

Pairwise comparisons performed using Tukey’s HSD test demonstrated significantly higher RTVs in the control group than in both experimental groups. The mean difference between the control and Flow.sil groups was 9.87 Ncm (95% CI: 4.24–15.49; *p* < 0.001), whereas the difference between the control and chlorhexidine groups was 7.74 Ncm (95% CI: 2.11–13.37; *p* = 0.005). No statistically significant difference was detected between the Flow.sil and chlorhexidine groups (mean difference = 2.13 Ncm; 95% CI: −3.50–7.75; *p* = 0.632) (Table 4).

Pairwise effect-size analysis further supported these findings. Very large effects were observed between the control and Flow.sil groups (Cohen’s d = 1.411) and between the control and chlorhexidine groups (Cohen’s d = 1.347). In contrast, the difference between the Flow.sil and chlorhexidine groups corresponded to a small effect size (Cohen’s d = 0.342), indicating substantial overlap between these groups (Table 5).

Outlier screening identified one potential outlier in the control group (46.70 Ncm) and one in the Flow.sil group (3.40 Ncm). Sensitivity analysis performed after excluding these observations yielded comparable results, with the overall ANOVA remaining statistically significant (F(2,40) = 8.39, *p* = 0.0006). These findings indicate that the observed group differences were robust and not dependent on individual extreme values.

## 4. Discussion

The present study investigated the influence of two antimicrobial sealing gels, namely a silicone-based sealing gel (Flow.sil) and a chlorhexidine-containing gel, on RTVs of implant–abutment screws following thermomechanical loading. The untreated control group demonstrated the highest RTVs after aging. Both gel-treated groups exhibited significantly lower RTVs than the control group, suggesting a less favorable mechanical response of the implant–abutment screw joint under the tested conditions. Although the Flow.sil group presented numerically lower RTVs than the chlorhexidine group, the difference between these two experimental conditions was not statistically significant. The primary hypothesis that the silicone-based sealing gel would provide higher RTVs following thermomechanical aging was rejected. Likewise, the assumption that the chlorhexidine and untreated control groups would exhibit comparable RTVs was not supported. Although the chlorhexidine group demonstrated lower RTVs as anticipated, the untreated control group unexpectedly yielded the highest RTVs among all experimental conditions.

Screw loosening has been extensively investigated in the implant dentistry literature and is generally attributed to insufficient preload and micromovements generated under functional loading conditions [23,24]. In cement-retained restorations, the cement layer has been reported to facilitate more favorable stress distribution by acting as a stress-absorbing medium between the crown and the abutment [23]. Furthermore, by compensating for minor interfacial discrepancies, the cement layer may contribute to enhanced prosthetic stability and improved mechanical performance of implant–abutment screw joint under occlusal loading [25]. In a previous investigation evaluating external connection implant systems, functional loads were reported to be transmitted directly to the abutment screw, resulting in stress concentration at the IAI and consequently increasing the risk of screw loosening or fracture [26]. In contrast, internal connection systems distribute functional loads through the internal connection walls via a mechanical interlocking mechanism, thereby reducing stress transfer to the screw and contributing to preload maintenance [27,28]. Therefore, a cement-retained restoration supported by a vertically aligned internal connection implant–abutment assembly was selected in the present study to minimize potential confounding mechanical variables and to allow a more isolated evaluation of the effects of screw surface treatment on post-aging RTVs. Increasing abutment collar height has been associated with greater torque loss following cyclic loading, presumably owing to an increase in the effective cantilever length, as reported by Siadat et al. [29]. In the present study, all specimens were standardized using abutments with a 2-mm collar height, thereby eliminating collar height as a potential confounding variable.

Because residual preload cannot be measured directly under most experimental conditions, RTV is commonly used as a surrogate parameter for evaluating SJS after fatigue loading [19]. However, RTV represents an indirect measure of preload retention and may also be influenced by factors such as thread friction and interfacial interactions during screw loosening. Therefore, RTV findings should be interpreted as an indirect indicator rather than a direct measurement of preload. Although the primary outcome of the present study was the evaluation of post-aging RTV, future investigations may further strengthen the interpretation of these findings by incorporating matched non-aged control groups for each treatment condition or RTV measurements obtained immediately after the retorque interval in separate specimens, thereby enabling a more comprehensive assessment of the individual contributions of tightening, thermomechanical aging, and interfacial interactions to screw joint behavior. Since determination of RTV requires intentional loosening of the implant–abutment screw, investigations assessing this outcome are predominantly conducted under in vitro conditions rather than in clinical settings [1,7,30]. To simulate clinical function, thermomechanical loading protocols employing frequencies ranging from 1 to 19 Hz have frequently been used for in vitro studies [27]. Furthermore, a long-term clinical investigation reported that screw-related complications most commonly occurred within the first 10 months of function [31]. Accordingly, a loading frequency of 2 Hz and 750,000 thermomechanical cycles, corresponding to approximately three years of clinical occlusal function, were selected in the present study to simulate posterior loading conditions. Thermocycling alone has been reported to be insufficient for the assessment of screw loosening [32]. Therefore, both thermal cycling and mechanical loading were applied simultaneously in the present study to more closely simulate the oral environment. Occlusal loading was applied under an off-axis configuration in accordance with ISO 14801 recommendations, thereby reproducing a more clinically relevant loading scenario for the implant–abutment assembly [21,30].

Because the IAI represents a potential reservoir for microbial contamination, the use of antimicrobial agents within this region has gained considerable attention. However, concerns have also been raised regarding their potential to compromise SJS over time [3]. In the present study, the chlorhexidine gel group exhibited significantly lower RTVs than the untreated control group. Although RTVs were numerically higher in the chlorhexidine group than in the silicone-based gel group, the difference between the two experimental groups was not statistically significant. Consistent with the findings of the present study, Asli et al. (2017) reported higher rates of screw loosening in specimens treated with chlorhexidine gel [33]. The authors attributed this finding to the lubricating effect of the gel, which may reduce the coefficient of friction and consequently interfere with the generation of an effective preload [33]. Similarly, Shemtov et al. (2023) observed lower RTVs in the chlorhexidine gel group than in the untreated control group [34]. In contrast, other investigations have suggested that the use of 0.2% chlorhexidine may reduce the risk of screw loosening [1,35,36]. Koosha et al. (2020) observed the highest RTVs in specimens treated with chlorhexidine gel compared with those exposed to blood, saliva, and fluoride-containing artificial saliva [1]. Furthermore, several studies have reported no significant effect of chlorhexidine application on SJS [14,23].

Previous studies have evaluated chlorhexidine-containing agents at various concentrations, including 0.1% [14], 0.2% [1,35], and 1% [37]. In the present study, a 2% chlorhexidine gel was used, consistent with the protocol reported by Ozdiler et al. (2021) [38]. However, unlike the findings of the present investigation, those authors reported no significant differences between the chlorhexidine gel, silicone-based gel, and untreated control groups. This discrepancy may be related to differences in the aging protocol, as their study employed a lower loading frequency (1 Hz), lower loading force (50 N), and fewer loading cycles (500,000 cycles) than those used in the present study [38]. Collectively, the available evidence indicates that the effects of chlorhexidine-containing agents on SJS remain highly variable across studies. Such inconsistencies may be attributed to differences in screw design and material, implant–abutment connection geometry, comparator lubricants, chlorhexidine concentration, and thermomechanical aging protocols [23]. Furthermore, previous investigations have employed acrylic resin blocks [1], stainless-steel holders [15], and custom-manufactured titanium test assemblies as supporting structures [3]. While studies based on acrylic resin and stainless-steel models generally reported more favorable outcomes for chlorhexidine-treated specimens, Rathe et al. (2021) observed no significant influence of chlorhexidine on preload values using a custom titanium test assembly [3]. In contrast, implant–abutment assemblies in the present study were embedded in an epoxy resin model material with an elastic modulus similar to that of alveolar bone [39], allowing a more physiologically relevant simulation of load transfer and supporting structure deformation. These differences suggest that specimen design and supporting materials may contribute to the heterogeneity of the reported findings.

One possible explanation for these findings may be related to the settling effect occurring at the IAI. Owing to microscopic surface irregularities, complete contact between the mating surfaces is not achieved immediately after tightening. Under functional loading, gradual flattening of these surface asperities may result in preload loss and a corresponding reduction in RTV [23]. Since lubricating agents can alter frictional behavior at the interface, they may influence the magnitude of settling and the long-term maintenance of preload. Therefore, the lower RTVs observed in the chlorhexidine group may be attributable, at least in part, to alterations in the tribological conditions at the screw–implant interface during thermomechanical aging. As an indirect mechanical outcome, post-aging RTV inherently reflects the combined effects of torque-to-preload conversion during tightening, embedment relaxation, gel setting or redistribution, preload degradation during thermomechanical loading, and breakaway friction during reverse rotation, rather than the isolated contribution of any single mechanical mechanism. Accordingly, the mechanisms underlying the effects of chlorhexidine-containing agents on SJS remain incompletely understood. Therefore, further investigations incorporating different chlorhexidine concentrations, implant configurations, connection geometries, and loading conditions are warranted to establish more definitive conclusions regarding their influence on long-term mechanical stability using direct preload measurement techniques.

Contrary to the initial hypothesis, the silicone-based sealing gel group exhibited the lowest RTVs among all experimental groups. These findings suggest that application of Flow.sil to the abutment screw threads did not provide any apparent advantage in terms of post-aging RTV. The consistently lower RTVs observed in this group indicate that the silicone-based material failed to provide the anticipated mechanical benefit under the experimental conditions of the present study. Notably, these findings are consistent with a previous investigation that reported significantly greater percentage torque loss in KieroSeal-treated specimens than in both control and chlorhexidine-treated groups [40].

A variety of silicone-based sealing materials have been introduced to complement antimicrobial strategies at the IAI. In addition to limiting bacterial colonization, these materials have been proposed to provide a physical seal by filling the implant–abutment gap, thereby reducing bacterial penetration and microleakage [3]. However, silicone-based sealing agents are not chemically identical. GapSeal has been described as a highly viscous silicone matrix containing 5 wt% thymol [41], whereas Flow.sil is based on a polydimethylsiloxane matrix supplemented with thymol [10]. In contrast to the findings of the present study, Coelho et al. (2024) [42] reported higher RTVs following the application of a silicone-based sealing agent. However, the comparison group in that investigation consisted of abutment screws wrapped with PTFE tape, and the authors did not recommend PTFE application as a strategy for preventing screw loosening [42]. Conversely, a more recent study suggested that PTFE may contribute to preload maintenance when used under cyclic loading conditions alone [43]. These discrepancies highlight the potential influence of differences in experimental design and comparison protocols on the reported outcomes.

GapSeal does not undergo a setting reaction; therefore, the timing of screw tightening is not considered a critical factor when this material is used [22]. In contrast, KieroSeal undergoes chemical hardening, and delays in tightening have been reported to induce plastic deformation of the screw threads, potentially compromising complete seating of the abutment screw and resulting in lower RTVs [38]. Despite the growing interest in silicone-based sealing agents, studies evaluating their influence on screw loosening remain limited [7,22,43]. The available evidence is even more scarce for Flow.sil [10], for which data regarding its mechanical effects at the IAI are largely lacking. For this reason, Flow.sil was selected as a test material in the present study, and its performance was compared with that of an established antimicrobial agent.

In studies comparing KieroSeal and GapSeal, GapSeal has generally been reported to have no significant effect on preload [42], whereas KieroSeal has been associated with reduced preload-related outcomes [37]. Although the present study did not directly quantify the clamping force generated at the IAI, the lower RTVs recorded in the Flow.sil group appear to be in line with the observations of Biscoping et al. (2018), who reported less favorable mechanical performance following the application of a silicone-based sealing material [37]. Similar to KieroSeal, Flow.sil exhibits time-dependent material behavior associated with its setting process, which may influence the tribological conditions at the IAI during function. Therefore, it may be speculated that the lower RTVs recorded in the present study could be related to time-dependent alterations in the material following application. Nevertheless, given the compositional differences among commercially available silicone-based sealants, direct comparisons between individual products should be interpreted with caution.

In contrast to the low RTVs observed in the Flow.sil group in the present study, Katebi et al. (2024) [22] reported significantly higher RTVs following cyclic loading when GapSeal was injected into the implant cavity prior to abutment screw tightening. Their study included both a GapSeal and a control group and demonstrated a beneficial effect of the silicone-based sealing agent on preload maintenance. However, despite employing the same loading frequency, the authors used fewer loading cycles (500,000) and a lower load magnitude (75 N) than those applied in the present investigation [22]. Similar findings were reported in another study comparing control and GapSeal-treated specimens across three different implant systems [7]. The authors observed higher RTVs in the silicone-treated groups both before and after cyclic loading and additionally reported lower implant–abutment microleakage values for the Straumann system. Although the same implant brand was used in the present study, the discrepancy between our findings and those reported previously may be attributable to the substantially greater thermomechanical challenge applied in the current protocol, including approximately twice the number of loading cycles, the incorporation of thermal fluctuations, and the use of a silicone-based material with a different composition. Moreover, GapSeal and Flow.sil differ in both formulation and setting behavior, which may further limit direct comparisons between the two materials.

Among the limited number of studies evaluating Flow.sil, Smojver et al. (2022) [10] compared this material with GapSeal and Oxysafe gel with respect to microleakage prevention. Under static conditions, GapSeal demonstrated the most effective sealing performance, whereas Flow.sil did not achieve comparable results. Although microleakage was not evaluated in the present study, these findings further suggest that silicone-based sealing materials should not be considered a homogeneous group and may exhibit distinct behaviors depending on their composition and physicochemical characteristics. In this context, the lower RTVs observed in the Flow.sil group further emphasize that the performance of Flow.sil differs from that of GapSeal across different experimental outcomes. The lower RTVs recorded in the Flow.sil group may be related to differences in material composition, rheological characteristics, and tribological behavior at the IAI. Consequently, changes in frictional conditions, settling phenomena, or material consistency during thermomechanical aging may have contributed to the increased torque loss observed in the present study. However, parameters such as frictional behavior, rheological characteristics, degree of setting, and material distribution at the IAI were not directly evaluated. Therefore, these explanations should be interpreted as possible inferences based on the observed findings rather than experimentally confirmed mechanisms. Furthermore, the present findings should be interpreted exclusively with respect to the mechanical outcomes evaluated in this study. Since post-aging RTV and antimicrobial sealing performance represent distinct outcome measures, the present results do not permit conclusions regarding the overall biological–mechanical performance or overall clinical performance of the evaluated sealing agents. Moreover, further studies directly comparing different silicone-based sealing agents under standardized thermomechanical conditions are needed to clarify their respective effects on preload maintenance, reverse torque behavior, and long-term SJS.

Interestingly, the control group exhibited the highest RTVs following thermomechanical aging. Similar findings have been reported previously, where untreated control specimens demonstrated greater joint stability after cyclic loading than PTFE-treated specimens [44]. Likewise, Wu et al. (2017) reported a lower risk of screw loosening in the absence of lubricant application [15]. In addition, commercially pure Grade 4 titanium screws have been reported to be more susceptible to loosening than titanium alloy screws under functional loading conditions [45]. Previous investigations that observed lower RTVs in untreated control groups than in chlorhexidine-treated groups employed commercially pure Grade 4 titanium screws [36,46], whereas titanium alloy prosthetic screws were used in the present study. Given the reported differences in the mechanical behavior and loosening resistance of these materials, variations in screw alloy composition may have influenced reverse torque behavior, thereby contributing to the discrepancies between studies.

A possible explanation for the favorable RTV performance observed in the control group may be related to the preservation of direct metal-to-metal contact at the IAI. Relative sliding between contacting metallic surfaces during tightening and functional loading has been reported to induce surface wear, which may alter the frictional characteristics of the interface over time [47]. Consequently, the tribological conditions established in the absence of sealing or lubricating agents may differ from those occurring in treated groups, thereby influencing post-aging RTV, which may indirectly reflect changes in SJS. Furthermore, direct metal-to-metal contact may have resulted in more predictable frictional behavior during tightening and subsequent thermomechanical loading. Nevertheless, the precise relationship among interfacial wear, frictional behavior, and the resulting tribological conditions at the IAI remains incompletely understood, as these interrelated factors may collectively contribute to the observed RTV patterns through multiple concurrent mechanical processes. Future studies combining direct preload measurements using strain-gauged abutment screws or load-cell-based systems with comprehensive tribological characterization, including coefficient of friction analysis, surface profilometry, and scanning electron microscopy of the implant–abutment interface may provide a better understanding of the mechanisms underlying these findings.

Notably, RTVs exceeding the initial tightening torque of 35 Ncm were observed in several control specimens and only occasionally in the Flow.sil group, whereas no such observations were recorded in the chlorhexidine group. Although this observation may seem counterintuitive, similar findings have been reported under specific experimental conditions in previous in vitro studies [19,30,35]. A plausible explanation is the combined effect of settling, also referred to as embedment relaxation, and the progressive adaptation of microscopic surface irregularities at the IAI during thermomechanical loading. As the contacting surfaces become more closely adapted, a greater real area of contact may be achieved, thereby increasing the resistance encountered during reverse torque testing [19]. In addition, breakaway friction and interfacial adhesion may also have contributed to the elevated RTVs observed in some specimens. Consequently, RTVs may occasionally exceed the initially applied tightening torque in individual specimens. Nevertheless, further studies incorporating advanced surface characterization and more comprehensive mechanical analyses are needed to provide a better understanding of the mechanisms underlying this phenomenon.

The findings of the present study should be interpreted in light of certain limitations. Only a single implant system, implant–abutment connection design, cement-retained abutment configuration, and thermomechanical aging protocol were evaluated. In addition, crown seating was performed using finger pressure rather than a predefined seating load, which may have introduced a minor source of variability during the cementation procedure. In addition, although gel application was performed by the same operator using a consistent application technique, the amount of gel was not standardized gravimetrically or volumetrically. Therefore, minor variations in gel layer thickness or material displacement at the IAI cannot be completely excluded and may have contributed to within-group variability. Furthermore, preload was not measured directly, and the mechanisms underlying the observed differences in reverse torque behavior could not be investigated at the microscopic level. Accordingly, the post-aging RTV should be interpreted as an indirect measure of screw joint behavior, reflecting the cumulative influence of multiple mechanical factors rather than the isolated effect of any single process. The tested materials were intentionally applied to the abutment screw threads rather than injected into the implant cavity, as reported in several previous studies investigating GapSeal [7,22,43], because the primary objective was to evaluate their direct influence on screw joint mechanics. It should also be noted that, unlike GapSeal, which remains as a non-setting silicone matrix, Flow.sil exhibits time-dependent setting behavior. Therefore, direct application to the screw threads was considered more appropriate for evaluating its influence on screw joint mechanics while avoiding potential influences related to material setting within the implant cavity. Accordingly, the present findings should be interpreted within the context of this specific experimental approach. Furthermore, only one chlorhexidine concentration and one silicone-based sealing material were included. Future studies should evaluate different implant configurations, a broader range of sealing materials with quantitatively controlled application protocols, and loading conditions while incorporating complementary analytical techniques, such as direct preload measurements, surface characterization, scanning electron microscopy, or micro-computed tomography, to further clarify the tribological mechanisms associated with preload maintenance and SJS. In addition, further clinical investigations are warranted to determine whether the mechanical behavior observed under controlled laboratory conditions can be reproduced under long-term intraoral function.

## 5. Conclusions

Within the limitations of this in vitro study, application of both the chlorhexidine-containing gel and the silicone-based sealing gel to the abutment screw threads resulted in significantly lower RTVs following thermomechanical aging compared with the untreated control condition. Although no statistically significant differences were detected between the two sealing agents, the Flow.sil group exhibited the lowest RTVs among the experimental groups. These findings suggest that, under the present experimental conditions, direct application of antimicrobial and silicone-based sealing agents to the abutment screw threads was associated with greater torque loss than the untreated control condition and may have influenced the mechanical response of the IAI.

Under the experimental conditions of the present study, the untreated control condition demonstrated the lowest torque loss. Accordingly, the potential mechanical effects of antimicrobial sealing agents should be considered when selecting screw-sealing protocols. Given the complex relationship among frictional behavior, material characteristics, and screw joint mechanics, further research is needed to clarify the mechanical implications of antimicrobial sealing agents at the IAI.

## Figures and Tables

**Table 1:** Descriptive statistics of reverse torque values and torque loss percentages according to experimental groups following thermomechanical aging.

Outcome	Group	*n*	Mean	SD	Median	Min–Max	95% CI
RTV (Ncm)	Control	15	33.03	6.57	33.00	23.40–46.70	29.70–36.35
	Flow.sil Gel	15	23.16	7.39	23.90	3.40–36.10	19.42–26.90
	Chlorhexidine Gel	15	25.29	4.78	25.70	19.10–33.40	22.87–27.71
Torque Loss (%)	Control	15	5.64	18.77	5.71	−33.43–33.14	−3.86–15.14
	Flow.sil Gel	15	33.83	21.11	31.71	−3.14–90.29	23.15–44.51
	Chlorhexidine Gel	15	27.75	13.66	26.57	4.57–45.43	20.84–34.66

RTV, reverse torque value; SD, standard deviation; CI, confidence interval. RTVs are expressed in Ncm, and torque loss is expressed as a percentage of the initial tightening torque (35 Ncm). Negative torque loss values indicate RTVs exceeding the initial tightening torque and do not necessarily indicate an actual increase in preload.

**Table 2:** (**a**) Shapiro–Wilk normality test results and (**b**) Levene’s test results for homogeneity of variances.

Test	Outcome	Group	Statistic	*p* Value
(**a**)				
Shapiro–Wilk		Control	W = 0.946	0.469
	RTV	Flow.sil Gel	W = 0.906	0.117
		Chlorhexidine Gel	W = 0.925	0.226
(**b**)				
Levene’s	RTV	All groups	F = 0.237	0.790

RTV, reverse torque value; W, Shapiro–Wilk test statistic; F, Levene’s test statistic. Values of *p* > 0.05 indicate normal distribution and homogeneity of variances.

**Table 3:** One-way ANOVA results comparing reverse torque values among the experimental groups.

Outcome	F	df	*p* Value	η^2^	Effect Size
RTV	10.058	(2, 42)	0.0003	0.324	Large

RTV, reverse torque value; F, ANOVA F statistic; df, degrees of freedom; η^2^, eta-squared effect size. Statistical significance was set at *p* < 0.05. Statistically significant differences were observed among the experimental groups for RTV (*p* < 0.001).

**Table 4:** Tukey HSD post hoc comparisons for reverse torque values among the experimental groups.

Outcome	Comparison	Mean Difference	*p* Value	95% CI	Interpretation
	Control vs. Flow.sil Gel	9.87	<0.001	[4.24, 15.49]	*** Significant
RTV	Control vs. Chlorhexidine Gel	7.74	0.005	[2.11, 13.37]	** Significant
	Flow.sil Gel vs. Chlorhexidine Gel	2.13	0.632	[−3.50, 7.75]	Not significant

RTV, reverse torque value; CI, confidence interval. Significance levels: ** indicates *p* < 0.01; *** indicates *p* < 0.001.

**Table 5:** Cohen’s d effect size estimates for pairwise comparisons of reverse torque values.

Comparison	Cohen’s d	Interpretation
Control vs. Flow.sil Gel	1.411	Very large
Control vs. Chlorhexidine Gel	1.347	Very large
Flow.sil Gel vs. Chlorhexidine Gel	0.342	Small

According to Cohen’s *d* interpretation, values ≥ 1.30 were considered very large effects, whereas values < 0.50 were considered small effects.

## Data Availability

The data presented in this study are available on request from the corresponding author due to their potential use for future research purposes. The data are not publicly available due to their planned use in future research projects.

## Abbreviations

The following abbreviations are used in this manuscript:

RTV	Reverse torque value
ANOVA	Analysis of variance
IAI	Implant–abutment interface
SJS	Screw joint stability
PTFE	Polytetrafluoroethylene
CAD	Computer-aided design
CAM	Computer-aided manufacturing
LED	Light-emitting diode
MDP	10-methacryloyloxydecyl dihydrogen phosphate
SD	Standard deviation
CI	Confidence interval
df	Degrees of freedom
HSD	Honestly significant difference
IQR	Interquartile range
